# The Role of Plasma Transfusion in Massive Bleeding: Protecting the Endothelial Glycocalyx?

**DOI:** 10.3389/fmed.2018.00091

**Published:** 2018-04-18

**Authors:** Stefano Barelli, Lorenzo Alberio

**Affiliations:** ^1^Division of Haematology and Central Haematology Laboratory, CHUV, Lausanne University Hospital, University of Lausanne, Lausanne, Switzerland; ^2^Faculté de Biologie et Médecine, UNIL, University of Lausanne, Lausanne, Switzerland

**Keywords:** massive hemorrhage, shock, resuscitation, fresh frozen plasma, endothelium, glycocalyx

## Abstract

Massive hemorrhage is a leading cause of death worldwide. During the last decade several retrospective and some prospective clinical studies have suggested a beneficial effect of early plasma-based resuscitation on survival in trauma patients. The underlying mechanisms are unknown but appear to involve the ability of plasma to preserve the endothelial glycocalyx. In this mini-review, we summarize current knowledge on glycocalyx structure and function, and present data describing the impact of hemorrhagic shock and resuscitation fluids on glycocalyx. Animal studies show that hemorrhagic shock leads to glycocalyx shedding, endothelial inflammatory changes, and vascular hyper-permeability. In these animal models, plasma administration preserves glycocalyx integrity and functions better than resuscitation with crystalloids or colloids. In addition, we briefly present data on the possible plasma components responsible for these effects. The endothelial glycocalyx is increasingly recognized as a critical component for the physiological vasculo-endothelial function, which is destroyed in hemorrhagic shock. Interventions for preserving an intact glycocalyx shall improve survival of trauma patients.

The aim of this mini-review is to give an overview on plasma treatment in massive bleeding. We will briefly describe current pathophysiological concepts of vascular damage in hemorrhagic shock, summarize data on the use of plasma as a resuscitation fluid, and report experimental data suggesting a protective role of plasma on endothelial integrity.

## Trauma, Massive Hemorrhage, and Trauma-Induced Coagulopathy (TIC)

### Epidemiology and Definition of Massive Hemorrhage

The World Health Organization estimates that in the year 2000, 5 million people died of injuries, accounting for 9% of global annual mortality (1). After central nervous injury, massive hemorrhage represents the second-leading cause of death, being responsible for 30–40% of trauma-related mortality (1). Death can occur within 3–6 h by exsanguination from uncontrolled hemorrhage and one-third to half of the deaths occur before reaching the hospital (1, 2). Modern transfusion practices and blood supply make massive hemorrhage a potentially preventable cause of death in different settings (e.g., civilian or military trauma, surgery, post-partum). The benefit of blood component transfusion in the context of trauma has been discussed for many years but it is only since the retrospective study of Borgmann published in 2007 (3) that plasma transfusion has been recognized as a probable positive factor for survival. However, “survival bias” remains an unsolved pitfall of retrospective studies, not only for interpreting potentially causative factors related to survival (i.e., did the patient “survive because she received plasma transfusion” *or* did she “get plasma transfusion because survived long enough to receive it”?) but also for defining massive hemorrhage. In fact, the classical definition of massive hemorrhage is based on the number of packed red blood cells (PRBC) units transfused during the first 24 h after admission. High mortality rates during the first 24 h and rapid time course of massive hemorrhage make transfusion rate (e.g., ≥3 PRBC units/60 min) a more appropriate definition (4). In addition, data analysis from the PROMMT study enabled Rahbar et al. to identify those patients most likely to develop massive hemorrhage based on emergency admission variables, such as systolic blood pressure, heart rate, pH, and hemoglobin (5). This prospective observational study showed that transfusion with higher ratio of plasma to PRBC early in resuscitation is associated with an improved survival at 24 h (6). Specifically, adult trauma patients surviving beyond 30 min from admission and transfused with ≥1 unit of PRBC in the first 6 h and ≥3 units of PRBC during the first 24 h showed a significantly higher survival at 6 and 24 h, and 30 days when receiving plasma units and PRBC at a ratio of at least 1:1 (6, 7). Of note, such high plasma to PRBC ratios beyond the first 24 h was not associated with survival by day 30.

### Pathophysiological Concepts

The so-called acute trauma coagulopathy (ATC) (8) and TIC (9) have been conceptualized through different models, all converging to the key concept of «endothelial stress» (10–20), also named «endotheliopathy of trauma» (21, 22) or «shock-induced endotheliopathy» (23). The endothelium covering an area of about 5,000 m^2^ is one of the frailest and initial victims of massive hemorrhage (24). For instance, severe hypo-perfusion is associated with increased levels of circulating heparan sulfate, a component of the endothelial surface with anticoagulatory properties similar to heparin (25). Moreover, the co-existence of severe tissue injury, leading to high *in vivo* thrombin generation, and severe hypo-perfusion, leading to endothelial sufferance and thrombomodulin shedding, is complicated by circulating thrombin–thrombomodulin complexes culminating in systemic protein C activation and fibrinolysis (8, 9).

Several factors drive the system into a vicious circle: (1) on the one side, endothelial injury with enhanced vascular permeability leads to further loss of intravascular volume, hypovolemia, tissue hypoxia, and exacerbated shock and (2) on the other side, resuscitation-related blood dilution with acidosis and hypothermia (the classical iatrogenic triad) further impair vasculo-endothelial functions. In sum, massive hemorrhage means perfusion, oxygenation, coagulation, and metabolic failures.

## Plasma as a Resuscitation Fluid

### Plasma Type, Delivery, and Supply

Plasma sources and plasma processing have been developed during these last decades (18). Each preparation addresses and mitigates particular risks related to transfusion hazards: single donor fresh frozen plasma (FFP) vs pooled plasma or quarantine FFP vs pathogen-inactivated FFP to diminish the risk of transfusion-transmitted infections; FP24 (frozen within 24 h after donation) instead of standard FFP (frozen within 8 h after donation) to enable HLA testing and remove high risk units for TRALI; frozen plasma vs liquid or thawed plasma to extend storage duration; lyophilization formulas for rapid reconstitution. Study of the variability of coagulation factors and natural anticoagulants levels in different plasma preparations are summarized elsewhere (26). Of note, the factor V and factor VIII, known to be «labile» and critical in the evaluation of manufacture practice, show heterogeneous decrease during storage, depending on formulas. Several studies reveal how processing conditions (whole blood hold-time, storage duration/temperature before freezing, freezing mode, leucodepletion, pathogen inactivation, lyophilization) specifically influence coagulation factor levels, microparticles content, clot generation capacity and protein composition in plasma (27–30) and clotting factor stability after thawing (31). In massive hemorrhage management, logistical concerns, besides biological aspects such as type of plasma, FFP to PRBC ratio or functional monitoring of clot generation, matters as well. Time between trauma and transfusion, transport of plasma from blood bank to the clinical unit, mode of checking plasma unit before transfusion and provision of thawed/liquid plasma are most critical aspects in massive transfusion protocols (32–34).

Hence, one logistical challenge for blood bankers is plasma supply. Benefit from plasma transfusion in massive hemorrhage lead to growing use of «universal» but scarce AB plasma (35). At the same time, implementation of the «male policy» (plasma donation from male donors only) to improve the transfusion safety regarding risk of TRALI significantly restricts plasma availability. This demand/supply imbalance led the American Red Cross to consider the use of group A plasma to adult trauma patients (36). Novak et al. reported the experience of 12 trauma centers participating in the PROPPR study in managing plasma inventory to meet new guidelines issued by the American College of Surgeons in 2013 for trauma resuscitation (37). Rapid delivery is made possible by the selection of group A plasma with low titer anti-B, in addition to plasma formulations and thawing systems with short turnaround time.

### Benefit of Plasma Transfusion: Do Coagulation Factors Tell the Whole Story?

Since the time Borgman et al. demonstrated in 2007 that FFP transfusion in massive hemorrhage resulted in increased survival (3), researcher started to wonder which mechanisms may be responsible for this effect. The first hypothesis at hand would have been the correction of coagulopthy. However, plasma transfusion in the form of FFP cannot replace coagulation factor loss (38). Therefore, several publications aimed to investigate the benefit of plasma resuscitation on other pathophysiological variables, such as endothelial restoration (39–45).

## Endothelial Glycocalyx

### The Endothelial Glycocalyx Structure and Function

The endothelial glycocalyx is a thick (about 0.2–3.0 µm *in vivo*) (46, 47), negatively charged carbohydrate-rich layer coating the vascular endothelium (48–52). The glycocalyx *sensu stricto* is formed by cell membrane-bound sulfated proteoglycans, consisting of a core protein (e.g., transmembrane syndecan, membrane-bound glypican, or basement matrix-associated perlecan) with glycosaminoglycans side chains (e.g., heparan sulfate, hyaluronic acid, and chondroitin sulfate) (53), and cell membrane glycoproteins bearing sialoproteins (50, 53). Syndecan-1 (CD 138), a heparan sulfate containing proteoglycan, is one of the major constituents ensuring endothelial integrity (51). Under physiological conditions, positively charged soluble components (such as plasma proteins, enzymes, growth factors, cytokines, amino acids, and cations) and water are trapped in the glycocalyx forming an extended endothelial surface layer. The mesh formed by the gylcocalyx contains ~1 to 1.5 l plasma, which are in dynamic equilibrium with the flowing blood (48, 54, 55).

The glycocalyx has several recognized functions (Table 1) (49–52). In particular, it forms a physical barrier between blood and vessel wall (48, 56–60); it maintains blood fluidity by modulating the interactions of the endothelium with blood cells and proteins (50, 61–63); it regulates cell adhesion and vascular permeability (64); it creates a high intravascular colloid-osmotic gradient (65, 66); and it acts as a mechano-transducer, e.g., by sensing shear stress and inducing endothelial release of nitric oxide (60, 63, 67, 68). As it may be expected from its many functions, the disruption of the glycocalyx leads to several clinically relevant pathologies (48, 52, 61). In the following paragraph, we will discuss the effect of hemorrhagic shock and type of resuscitation fluid on the glycocalyx.

**Table 1:** Some recognized functions of the endothelial glycocalyx (48–50, 52, 69, 70).

Functions	Mechanisms	Reference
Barrier and filter	Protection from shear	(71)
	Exchange of water and solutes	(48)
	Sieve for plasma proteins	(57, 59)
	Uptake of low density lipoproteins	(63, 72)
	Repels red blood cells	(50)
Cell adhesion regulation	Prevents leukocyte adhesion	(56–58, 60, 73)
	Prevents platelet adhesion	(74)
Anticoagulation	Tissue factor pathway inhibitor	(48)
	Antithrombin	(50, 75)
	Thrombomodulin	(50)
Complement regulation	Complement factor H binding	(76)
Colloid-osmotic gradient	Absorption of albumin and smaller solutes	(65, 71)
Mechano-transducer	Nitric oxyde production	(50, 68, 77)
	Prostacyclin production	(48)
Inter-endothelial communication	Regulation of endothelial gap junctions	(78, 79)

### Hemorrhagic Shock, Endothelial Glycocalyx, and Resuscitation Fluid

Shedding of endothelial glycocalyx components has been shown to occur in response to, e.g., ischemia and hypoxia (80), reactive oxygen species (81), inflammation and sepsis (82), and trauma-related sympatho-adrenal activation (83). As recently reviewed by Becker et al., loss of glycocalyx appears to be mediated by “sheddases,” such as matrix metalloproteases, heparanases, hyaluronidases, and proteases (60) and to be responsible for endothelial inflammatory changes and vascular hyperpermeability (51, 64). In hemorrhagic shock, loss of the endothelial glycocalyx correlates with a dismal outcome. For instance, human studies indicate that in trauma patients with severe bleeding, high levels of syndecan-1 on admission (≥40 ng/ml) correlate with the extent of tissue damage, laboratory indicators of ATC and, in particular, mortality (84–86). Rahbar et al. showed that high circulating syndecan-1 levels correlate with increased vascular permeability (87). Since plasma-based resuscitation appears to exert a beneficial effect on survival (6, 7, 88), the question is whether plasma as a resuscitation fluid may have an impact on the endothelial glycocalyx and, therefore, potentially on vascular integrity and function.

Investigations in animal models may help framing a working concept (Table 2). Kozar et al. (40) employed a pressure-controlled model of hemorrhagic shock. Rats were bled to a mean arterial pressure of 30 mmHg for 90 min then resuscitated with either lactated Ringer’s solution (LR) or fresh plasma to a mean arterial pressure of 80 mmHg. These animals were compared to shams (all procedures without bleeding) and positive controls (hemorrhagic shock without resuscitation). The authors found that (1) hemorrhagic shock is associated with a significant shedding of the endothelial glycocalyx, as indicated by circulating syndecan-1 levels, cell surface expression of syndecan-1, and electron microscopy imaging; (2) loss of the endothelial syndecan-1 correlates with the extent of lung injury, as assessed by alveolar wall thickness, capillary congestion, and cellularity; (3) resuscitation with plasma partially restores the endothelial glycocalyx while LR cannot, as assessed by electron microscopy on post-capillary venules obtained from the small bowel mesentery and by syndecan-1 expression in the lung; (4) the endothelial glycocalyx appears to be restored within 3 h after plasma resuscitation; (5) a clinically potential beneficial effect of plasma is suggested by the observations that plasma resuscitation required significantly less volume to maintain the mean arterial pressure at 80 mmHg compared to LR, and by the fact that plasma reduced lung injury while LR resuscitation increased it (40). These observations were expanded by the work of Torres et al. (42). In their model, a 40% blood volume hemorrhage was induced in rats. After 30 min of shock, animals were resuscitated with LR, hydroxyethyl starch (HES) or FFP, and compared to sham and hemorrhage without resuscitation. First, the authors confirmed that the endothelial glycocalyx is significantly damaged by the hemorrhagic shock and can be restored only with FFP, as assessed by circulating syndecan-1 levels and glycocalyx thickness. Second, a clinically beneficial effect of plasma-based resuscitation was indicated by the fact that FFP corrected metabolic acidosis significantly better than LR and HES, as assessed by pH, base excess, and lactate. This was associated with an improved microcirculation and a lesser degree of hemodilution by FFP compared to LR and HES (42). This latter point was also observed by a recent publication of Nelson et al. (89), who demonstrated that resuscitation with FFP resulted in a circulating volume expansion equaling the volume of blood loss, while circulating volume expansion by Ringer’s acetate was less effective.

**Table 2:** Studies^a^ investigating endothelial integrity through plasma exposition in HS conditions.

Reference	Experimental models	Types of plasma	Main results
Pati et al. (39)	Studies on HUPECs monolayers (hypoxia-induced permeability) with assessment of EC permeability (FITC-Dextran) after FFP treatment, comparing FFP stored for 0 vs FFP stored for 5 days *In vivo* studies on rat model of HS for testing capacity of FFP (comparing FFP stored for 0 vs for 5 days) to restore MAP	Human FFP (ABO blood types, same donor or pooled from three donors, thawed-aliquoted-stored at 4°C for 0 or 5 days before use)	Day 0 FFP inhibits EC permeability; day 5 FFP demonstrated a diminished capacity to inhibit EC permeability Day 0 FFP, but not day 5 FFP, restores blood pressure to baseline
Kozar et al. (40)	Studies on a rat model of HS, comparing effect of LR vs fresh plasma resuscitation with assessment of endothelial glycocalyx on mesenteric vessels (electronic microscopy), relative expression level of syndecan-1 (QRT RT PCR) and cell surface expression of syndecan-1 (immunostaining) in lung tissue	Fresh plasma (not otherwise specified)	Glycocalyx is partially restored by plasma resuscitation Syndecan-1 expression in lung is enhanced by plasma Lung injury is lessened by plasma resuscitation
Haywood-Watson et al. (86)	Studies on HUVECs monolayers (hypoxia-induced permeability) with assessment of VE-cadherin and syndecan-1 expression (immunofluorescence), topographical properties (AFM), permeability (FITC-Dextran) after LR vs FFP treatment Patients admitted to ICU for shock, resuscitation with plasma, syndecan-1 and cytokines measurements	FFP (not otherwise specified)	Vascular integrity is disrupted by shock but mitigated by FFP FFP hastens syndecan-1 restoration compared to LR Injured patients in shock shed syndecan-1; syndecan-1 correlates with specific inflammatory cytokines
Torres et al. (42)	Studies on a rat HS models comparing effect of LR/HS vs fresh plasma resuscitation with studies on blood samples (including thromboelastometry) and on endothelium (glycocalyx thickness measurements by fluorescent dye-exclusion method)	FFP defined as plasma frozen within 6–8 h of collection and stored at −20°C, prepared by separation form whole blood collected on donor rats	Restoration of coagulation function by a small-volume resuscitation with FFP in contrast to resuscitation with LR/HS groups
Peng et al. (41)	Studies on HUPECs monolayers (VEGF-A165-induced permeability) with assessment of EC permeability (TEER/ECIS and FITC-Dextran) and leukocyte-endothelial binding Mouse model of HS and trauma comparing effect of LR vs FFP resuscitation with *in vivo* studies (MAP monitoring, measurement of syndecan-1 in plasma) and *in vitro* studies on harvested lungs: vascular permeability (intravenous fluorescent dye extravasation), infiltration of neutrophils (MPO immunofluorescence staining and activity), syndecan-1 detection (anti-syndecan-1 antibody)	Human FFP used in both *in vitro* and *in vivo* studies (frozen within 8 h after donation, kept frozen until the day of experiment and used within 1–2 h of thaw)	In HUPECs monolayers, FFP compared with LR reduces pulmonary endothelial hyper-permaeability and leukocyte binding In mouse HS models, FFP and LR similarly restore MAP. FFP mitigates lung hyper-permeability, reduces lung inflammation, increases lung syndecan-1, and reduces syndecan-1 shedding compared with LR resuscitation
Wataha et al. (44)	Studies on HUVECs and PECs monolayers (VEGF-A165-induced permeability) comparing effect of FFP, SD-FFP, SDP (controls: LR/HS) with assessment of EC permeability (FITC-Dextran), WBC binding assay (fluorescent labeling), surface adhesion molecules/integrin expression (flow cytometry) and VE-cadherin/β-catenin mobilization to cell surface (staining)	Human FFP (frozen at −20°C, thawed at 37°C and used on day 0–1 of thaw)SDP defined as pooled liquid plasma that has been dehydrated by means of spray drying and reconstituted citric acid and monobasic sodium phosphate (SD-FFP being the starting material)	FFP, SD-FFP, and SPD equivalently inhibit vascular permeability, ensures EC adherens junctions integrity and endothelial WBC binding Lack of difference between FFP and SD-FFP and between SD-FFP and SDP indicating that solvent-detergent treatment and spray drying do not affect the ability of plasma product to modulate endothelial function
Potter et al. (45)	Studies on HUVECs monolayers (VEGF-A165-induced permeability), comparing FFP and SDP (controls: LR) by testing endothelial permeability (TEER/ECIS), cytokine production in EC and gene expression Mouse model of HS comparing FFP and SDP (controls: LR) with *in vivo* studies (MAP and BE monitoring) and measurement of EC adherent junctions stability (immunofluorescence and histological staining) on harvested lungs	FFP obtained from human donors plasma by apheresis collection, used freshly thawed (same day of thaw)SDP from multidonor plasma (more than 150 type AB donors)	On HUVECs monolayers, FFP and SDP decrease endothelial permeability, induce similar patterns of gene expression and cytokines production in EC In mouse HS models, SDP and FFP equivalently correct MAP and BE, reduce pulmonary vascular leak, equivalently inhibit leukocyte infiltration and breakdown of endothelial adherens and tight junctions
Torres Filho et al. (90)	Rat model of HS for studying quantitatively the relationship between plasma biomarkers and changes in microvascular parameters, including glycocalyx thickness after resuscitation with FWB, PRBC, FFP, 5% albumin, or crystalloids (RL, NS, and HTS)	FWB (3.2% citrate, stored at 4°C, used with 24 h), PRBC (used within 48 h), and FFP (frozen within 6–8 h of collection, stored at −80°C for up to 1 year) all from donor rats	Changes in glycocalyx thickness (and microvascular permeability) negatively (positively) correlated with changes in plasma levels of syndecan-1 and heparane sulfate FWB and FFP, but neither colloid or crystalloid resuscitation, support vascular stabilization by reconstitution of the endothelia glycocalyx after HS
Diebel et al. (91)	HUVEC lined microfluidics model for studying endothelial cell activation/injury and glycocalyx barrier function after simulation of HS by treatment with epinephrine and hypoxia reoxygenation	5% human plasma perfused immediately following treatment or after a 3 h delay	“Early” plasma mitigates glycocalyx degradation and inflammatory prothrombotic endothelial response
Pati et al. (92)	Studies on HUVECs monolayers (VEGF-A165-induced permeability), comparing FFP and LP (controls: LR or no treatment) by testing EC permeability (FITC-Dextran), EC resistance (TEER/ECIS), VE-cadherin/β-catenin mobilization to cell surface (staining), leukocyte-binding (fluorescent labeling) Mouse model of HS comparing FFP and LP (controls: LR or no treatment) with assessment of inflammation (MPO staining), vascular permeability (dye extravasation) and tissue edema (wet-to-dry weight ratio)	Human FFP (male donors O+) thawed and used freshly (day 0 of thaw) LP defined as lyophilized plasma (male O+) reconstituted in buffer	On HUVECs monolayers, FFP and LP decrease endothelial permeability, preserve EC adherens junctions, attenuate EC-leukocyte-binding In mouse HS models, LP and FFP reduce pulmonary vascular permeability, edema, and inflammation

*AFM, atomic force microscopy; BE, base excess; EC, endothelial cell; ECIS, electric cell-substrate impedance system; FFP, fresh frozen plasma; FITC, fluorescein isothiocyanate-conjugated; FWB, fresh whole blood; HES, hydroxyethyl starch; HS, hemorrhagic shock; HTS, hypertonic (3%) sodium chloride; HUPEC, human pulmonary endothelial cell; HUVEC, human umbilical vein endothelial cell; LP, lyophilized plasma; LR, lactated ringers; MAP, mean arterial pressure; NS, normal saline; PEC, pulmonary endothelial cells; PRBC, packed red blood cells; QRT RT PCR, quantitative real-time reverse-transcription polymerase chain reaction; SD, solvent detergent; SDP, spray-dried plasma; TEER, trans-endothelial electrical resistance; VE-cadherin, vascular endothelial cadherin; WBC, white blood cell*.

*^a^Studies identified by searching the terms “glycocalyx, haemorrhagic shock, plasma” on PubMed and secondary references*.

The pulmonary effects of hemorrhagic shock and resuscitation fluids were addressed by Peng et al. (41). They investigated pulmonary endothelial inflammation and hyper-permeability employing a coagulopathic mouse model of hemorrhagic shock and trauma. Mice were bled to a mean arterial pressure of 35 ± 5 mmHg for 90 min (93) and subsequently resuscitated over 15 min with either LR (at 3× shed blood volume) or FFP (at 1× shed blood volume). Resuscitated animals were compared to shams (all procedures without shock) and positive controls (hemorrhagic shock without resuscitation). Major findings were as follows: (1) lung permeability, assessed *in vivo* by the extravasation of a fluorescent dextrane or Evan’s blue, was significantly increased after hemorrhagic shock compared to shams, and FFP resuscitation was significantly more effective than LR in preventing/correcting shock-induced pulmonary hyper-permeability; (2) similarly, lung inflammation, assessed by detecting myeloperoxidase which reflects neutrophils infiltration, significantly increased after hemorrhagic shock and was lessened by FFP resuscitation; (3) shock-induced loss of pulmonary syndecan-1 was most efficiently prevented by resuscitation with FFP. Of note, similar results on pulmonary inflammation and permeability were reported by Potter et al. employing FFP and spray-dried plasma (SDP) (45).

A recent publication by Torres Filho et al. (90) employing a rat model of hemorrhagic shock showed that (1) syndecan-1 and heparan sulfate represent valuable biomarkers of glycocalyx shedding and (2) fresh whole blood and FFP support vascular stabilization by reconstitution of the endothelial glycocalyx (see Table 2).

### Syndecan-1 as a Key Mediator of Plasma’s Effect

A key question is which plasma component may exert a beneficial effect on the glycocalyx. *In vitro* experiments have shown that FFP enhances pulmonary endothelial syndecan-1 expression in a time- and dose-dependent manner (94). A key role for syndecan-1 is supported by *in vivo* experiments as well. Utilizing the model of trauma-hemorragic shock described by Peng (41), Wu et al. investigated the pulmonary response to the type of resuscitation fluid (FFP vs LR) in wild-type and *Syndecan* gene knock-out (Sdc1^−/−^) mice (94). They found that the inability to synthesize syndecan-1 abrogated the protective effect observed with plasma. In particular, they demonstrated that in absence of syndecan-1 synthesis: (1) the ability of FFP to mitigate the increase in lung permeability induced by hemorrhagic shock was abrogated; (2) FFP lost its ability to dampen the shock-induced increase of pulmonary neutrophil infiltration; and (3) FFP lost its protective effect on histopathologic signs of lung injury. Similar results have been reported by Ban et al. with an animal model of gut injury and inflammation after hemorrhagic shock (95).

### Plasma: Coagulation Factors or Other Components?

Intriguingly, a major plasma component that may play a role in preserving endothelial integrity appears to be albumin. While loss of circulating albumin correlated with loss of the glycocalyx and increased fluid extravasation (96), albumin supplementation attenuated glycocalyx shedding and reduced interstitial edema in a guinea pig heart model of cold ischemia (97). Kheirabadi et al. (98) studied the role of albumin in a model of uncontrolled hemorrhage. Rabbits were subjected to a splenic injury. Ten minutes after injury, at a mean arterial pressure less than 40 mmHg, the rabbits received equal volumes (15 ml/kg) of rabbit plasma, HES, or 5% human albumin, targeting a mean arterial pressure of 65 mmHg. The authors observed that: (1) onset of resuscitation initiated additional bleeding and total blood loss did not differ among the three groups; (2) thromboelastography revealed a faster and stronger clot formation in the plasma and albumin groups compared to HES; (3) shock indices were increased in all three groups but less in the albumin one; (4) the albumin group had the highest survival rate (8 out of 9 rabbits) compared to plasma and HES (both 4/10), and positive controls (1/9). This apparent beneficial role of albumin, if confirmed in further studies, may be related to its ability to attenuate neutrophil adhesion to the endothelium and other anti-inflammatory properties, its scavenging and buffering capacity, its potential to enhance nitric oxide production and stabilize glycocalyx (50, 60, 99). However, a recent publication showed that a four-factor prothrombin complex concentrate (containing vitamin K-dependent coagulation factors and several other plasma proteins) and FFP but not albumin inhibit vascular permeability in an *in vivo* mice model (100). Thus far, it is not known which soluble factor present in the factor concentrate might be responsible for its beneficial effect (100).

As of coagulation factors, despite a current of thought supporting the use of fibrinogen in massive bleeding, we are not aware of publications investigating its impact on glycocalyx and endothelial functions. A recent work observed a U-shaped association between initial fibrinogen concentration in major bleeding and in-hospital mortality, with similar rates of increased mortality for fibrinogen levels <1 g/l and >4 g/l (101). A possible explanation for the negative effect of higher fibrinogen levels is offered by *in vitro* data, suggesting that fibrin promotes endothelial transmigration of neutrophils and inflammation (102).

As of other plasma proteins, adiponectin is an interesting candidate (103). Adiponectin is produced in adipocytes and has been shown to have anti-inflammatory properties and to prevent cytokine-induced endothelial cell hyper-permeability (104–106). Employing a mouse model, Deng et al. demonstrated that (1) hemorrhagic shock leads to a significant decrease of adiponectin levels and a disruption of the lung vascular barrier function; (2) plasma resuscitation improves adiponectin levels and reverses lung injury; (3) the beneficial effect of plasma-based resuscitation is abolished by immunodepletion of adiponectin; and (4) it is restored when plasma was replenished with adiponectin (103). These findings suggest that adiponectin may be an important component contributing to a vasoprotective effect of plasma-based resuscitation.

In sum, several animal studies suggest that early use of plasma in hemorrhagic shock may exert a clinically significant beneficial effect by preserving or even restoring the glycocalyx layer and, therefore, maintaining critical endothelial functions. This appears to be due to the ability of a plasma component to lessen endothelial inflammatory response, possibly by limiting neutrophil adhesion. As of today, it is not known which plasma components are responsible for these effects, which impact plasma processing may exert on them, and which might be the dose–effect relationship.

### Human Studies

From a clinical point of view, the key question is whether early resuscitation of hemorrhagic shock with plasma is truly able to improve vasculo-endothelial function and survival. As a proof of principle, a small study in non-bleeding critically ill patients demonstrated that plasma transfusion decreased syndecan-1 and factor VIII levels, suggesting an endothelial stabilizing effect (107). To our knowledge, the only human study prospectively investigating the effect of early plasma-based resuscitation in humans is the COMBAT study (108). In this prospective randomized trial, casualties are treated with 2 units of FFP (thawed in the ambulance) vs conventional crystalloids as initial pre-hospital resuscitation. The study aims to verify whether a “plasma first” resuscitation strategy might be able to (1) attenuate acute traumatic coagulopathy; (2) improve metabolic recovery; (3) decrease blood component transfusion; (4) reduce the incidence of acute lung injury and multiple organ failure; (5) decrease mortality at 24 h or 28 days. According to www.clinicaltrials.gov, the study has been closed after having enrolled 144 patients as per protocol. Results are eagerly awaited.

In conclusion, plasma as early resuscitation fluid for massive hemorrhage appears to exert beneficial effects improving patient survival. Experimental data suggest that this may be related to its ability to preserve endothelial glycocalyx structure and function. We think that these fascinating data shall be confirmed in prospective randomized clinical trials and the mechanisms underlying these effects shall be revealed in order to develop more targeted treatments.

## Author Contributions

Both authors discussed the literature, wrote the manuscript, and approved the final version.

## Conflict of Interest Statement

The authors declare that the research was conducted in the absence of any commercial or financial relationships that could be construed as a potential conflict of interest.
